# Tooth Loss Is Associated with Disease-Related Parameters in Patients with Rheumatoid Arthritis and Ankylosing Spondylitis—A Cross-Sectional Study

**DOI:** 10.3390/jcm10143052

**Published:** 2021-07-09

**Authors:** Gerhard Schmalz, Markus Bartl, Jan Schmickler, Susann Patschan, Daniel Patschan, Dirk Ziebolz

**Affiliations:** 1 Department of Cariology, Endodontology and Periodontology, University of Leipzig, 04103 Leipzig, Germany; gerhard.schmalz@medizin.uni-leipzig.de (G.S.); markus.bartl95@gmx.de (M.B.); schmickler.jan@gmail.com (J.S.); 2 Department of Cardiology, Angiology and Nephrology, Klinikum Brandenburg, Medizinische Hochschule Brandenburg, 16816 Brandenburg, Germany; spatschan@gmail.com (S.P.); d.patschan@gmail.com (D.P.)

**Keywords:** rheumatoid arthritis, periodontitis, ankylosing spondylitis, disease activity, tooth loss

## Abstract

Background: The aim of this cross-sectional study was to investigate potential associations between periodontal inflamed surface area (PISA) and tooth loss with disease-related parameters in patients with rheumatoid arthritis (RA) and ankylosing spondylitis (AS). Methods: Patients who attended the Department of Nephrology and Rheumatology, University Medical Centre Goettingen, Germany, were included. The oral examination comprised the detection of the number of remaining teeth and periodontal condition based on staging and grading matrix. Based on periodontal pockets with positive bleeding on probing, the periodontal inflamed surface area (PISA) was determined. Disease related parameters were extracted from the patients’ records. Results: In total, 101 (RA) and 32 participants (AS) were included. Patients with RA had 22.85 ± 4.26 and AS patients 24.34 ± 5.47 remaining teeth (*p* < 0.01). Periodontitis stage III and IV was present in 91% (RA) and 81.2% (AS) of patients (*p* = 0.04). Associations between PISA and disease-related parameters were not found in both groups (*p* > 0.05). In RA, a higher age (*p* < 0.01), C-reactive protein (*p* = 0.02), disease activity (*p* < 0.01) and prednisolone intake (*p* < 0.01) were associated with fewer remaining teeth. In AS, a higher age (*p* = 0.02) and increased Bath Ankylosing Spondylitis Metrology Index (*p* = 0.02) were associated with a lower number of remaining teeth. Conclusions: Tooth loss is associated with disease activity, especially in RA individuals. Dental care to prevent tooth loss might be recommendable to positively influence oral health condition and disease activity in RA and SA patients.

## 1. Introduction

Rheumatic diseases, e.g., rheumatoid arthritis (RA) and ankylosing spondylitis (AS), can be related to different oral manifestations, whereby periodontal inflammation and burden are two of the most highlighted conditions [1]. Above all, RA, which primarily is an autoinflammatory disease affecting the joints, is associated with periodontitis [2,3,4]. It has been shown that patients suffering from RA had more severe periodontitis and a higher risk for tooth loss [5,6]. On the other hand, periodontal therapy was found to positively influence disease activity and biochemical markers of RA [7]. However, the supposed bidirectional relationship between periodontitis and RA still appears not evident within the available literature, in which a significantly worse periodontal condition and inflammation in patients with RA were not confirmed by recent systematic reviews and meta-analyses [3,4]. Similarly, a large cohort study by this working group found worse periodontal health in RA individuals but did not confirm clinical findings supporting a causal relationship between the two diseases [8]. Therefore, it can be concluded that more studies are needed in this field [4].

Moreover, AS, an autoinflammatory disease affecting the spinal joints, is associated with periodontal diseases [9,10]. It has been demonstrated that periodontal inflammation and disease burden are associated with disease-specific markers and complaints of patients suffering from AS [9,10]. However, the causality between the two diseases is still questionable, making more clinical studies necessary [11].

In the context of a deeper understanding of rheumatic diseases and their interrelationship with periodontitis, one diagnostic parameter might be of interest: the periodontal inflamed surface area (PISA). This parameter is able to quantify the inflammatory periodontal burden by determining the inflamed periodontal surface [12,13]. As this parameter displays the current periodontal inflammation, an association with rheumatic disease-related parameters might hint at an interrelationship between periodontal inflammation and the disease severity of rheumatic disease. One previous study examined the PISA of patients with RA and found it to be associated with disease activity and with the clinical response to disease modifying antirheumatic drugs [14]. More studies regarding the PISA of patients with RA or AS are not yet available. In particular, the current classification of periodontal diseases, i.e., the staging and grading matrix [15], has not been previously considered in this context.

Accordingly, this current cross-sectional study aimed to assess PISA in patients with RA and AS and to investigate whether PISA would be associated with rheumatic disease-related parameters in these individuals. Moreover, the number of remaining teeth and its potential association with disease-related parameters in RA and AS patients was examined. It was hypothesized that a higher PISA and fewer remaining teeth would be associated with increased disease activity and severity of RA and AS, respectively.

## 2. Methods

### 2.1. Study Design

This study was composed based on two cross-sectional studies, which examined patients with RA and AS regarding their periodontal condition. For this current cross-sectional examination, patients were selected by previously defined inclusion and exclusion criteria. All study protocols were reviewed and approved by the Ethics Committee of the medical faculty of the Georg-August-University Goettingen, Germany (application No. 14/2/13). All patients were informed in writing and within doctor-patient consultation and gave their informed consent for participation.

### 2.2. Study Participants

During their routine outpatient or stationary appointment at the Department of Nephrology and Rheumatology, University Medical Centre Goettingen, Germany, patients were consecutively recruited. Thereby, patients with a diagnosis of RA, made by an experienced rheumatologist according to the American College of Rheumatology and the European League Against Rheumatism (ACR/EULAR) [16], and patients with AS, diagnosed according to the modified New York criteria [17], were included in the study. The inclusion criteria for the current study were as follows: diagnosis of RA or AS described above, age between 18 and 79 years, complete clinical disease-related parameters and blood values and voluntary informed consent for participation. The following exclusion criteria were defined:

- Presence of comorbidities which may influence periodontal outcome, e.g., diabetes mellitus, renal diseases or cardiovascular diseases;
- Poor general health, making full periodontal examination impossible;
- Pregnancy or lactation;
- Immunosuppressive medication besides rheumatic disease-related medication (e.g., in case of organ transplantation);
- Infectious diseases, e.g., HIV or hepatitis;
- Addiction.

The general and disease-related parameters age, gender, smoking habits (smoker: currently smoking, non-smoker: currently no smoking), disease duration (date of first diagnosis), C-reactive protein in blood (CRP), medication (prednisolone, methotrexate and/or biologicals), number of swollen and/or painful joints and duration of morning stiffness were extracted from the medical records. For RA group, current “Disease Activity Score” of the RA (DAS 28-ESR) and levels of the antibodies against cyclic citrullinated peptide (aCCP) were recorded. For AS group, Bath Ankylosing Spondylitis Disease Activity Index (BASDAI), Bath Ankylosing Spondylitis Metrology Index (BASMI), Bath Ankylosing Spondylitis Functional Index (BASFI) and Bath Ankylosing Spondylitis Patient Global Score (BAS-G) were extracted.

### 2.3. Clinical Examination

Two experienced and calibrated dentists (kappa > 0.8) performed all clinical examinations. The oral cavity was investigated visually to record the number of remaining teeth, differentiated into number of remaining molars/premolars and remaining front teeth. Furthermore, patients received a complete periodontal examination, including periodontal probing depth (PPD) and bleeding on probing (BOP: positive), and clinical attachment loss (CAL) was assessed at 6 measurement points per tooth using a millimeter-scaled periodontal probe. Based on these findings, periodontitis was diagnosed based on the staging (I-IV) and grading matrix (A-C) [15]. Additionally, the periodontal inflamed surface area (PISA) was estimated to quantify inflamed periodontal pockets, as described by Nesse et al. [12] No information was available on any previous periodontal therapy, (regular) supportive periodontal care or recent intake of antibiotics at the time of oral examination. For the current study, radiographs were neither taken nor available to assess radiographic bone loss.

### 2.4. Statistical Analyses

All statistical analyses were performed with SPSS for Windows. Both groups (RA and AS) were analyzed separately and, whenever possible and reasonable, compared to each other. Metric variables were tested for normal distribution by Kolmogorov–Smirnov-test, showing non-normal distribution of the parameters (*p* < 0.05). Thus, nonparametric tests for non-normal distributed samples were used. Comparing two independent, non-normal distributed samples, Mann–Whitney U test was applied. More than two independent, non-normal distributed samples were compared by Kruskal–Wallis test. Categorical data were analyzed by chi-square or Fisher’s test, respectively. Significance level was set at *p* < 0.05.

## 3. Results

### 3.1. Patients

Within the RA group, 101 patients with a mean age of 57.48 ± 10.03 and consisting of 18.8% males were included. The AS group consisted of 32 participants, including 56.3% males and having a mean age of 48.37 ± 16.77 years. Different disease-related parameters and information regarding medication are shown in Table 1.

### 3.2. Periodontal Findings

Patients with RA had 22.85 ± 4.26 remaining teeth, which was less than AS patients 24.34 ± 5.47 (*p* < 0.01). The number of teeth with CAL ≥ 5 mm (*p* < 0.01) and number of teeth with PPD ≥ 5 mm (*p* = 0.02) were significantly higher in RA, while AS showed more BOP-positive pockets (*p* < 0.01). Periodontitis staging revealed more severe periodontitis in RA individuals (*p* = 0.04), while AS patients had a higher amount of grade C (*p* = 0.03). PISA was 564.69 ± 397.20 (RA) and 614.43 ± 357.12 (AS; *p* = 0.35), respectively (Table 2).

### 3.3. Associations between PISA and Disease-Related Parameters

In the RA group, smoking was associated with a higher PISA (*p* = 0.03). Associations between PISA and disease-related parameters were not found in the RA group (*p* > 0.05; Table 3). In AS individuals, no significant associations were detected between PISA and disease-related parameters, too (*p* > 0.05; Table 4).

### 3.4. Associations between Number of Remaining Teeth and Disease-Related Parameters

In RA patients, a higher age was associated with fewer remaining teeth (*p* < 0.01). Furthermore, higher CRP (*p* = 0.02), higher DAS28-ESR (*p* < 0.01) and medication with prednisolone (*p* < 0.01) were associated with fewer remaining teeth (Table 5). In the AS group, a higher age (*p* = 0.02) and increased BASMI (*p* = 0.02) were associated with a lower number of remaining teeth (Table 6).

## 4. Discussion

Summary of the main results: RA and AS patients suffered from a high prevalence of periodontitis stage III and IV. Thereby, RA individuals had worse periodontal conditions and fewer remaining teeth than AS individuals. PISA was not associated with any rheumatic disease-related parameters in both RA and AS groups. Fewer remaining teeth were associated with age, CRP, DAS-28 and prednisolone medication in the RA group and with age and BASMI in the AS group.

Interpretation of the results and comparison with the literature: A high prevalence of periodontal diseases in patients with rheumatic diseases, especially RA and AS, is well documented in the literature [2,9]. Thereby, it is conspicuous that no individual was periodontally healthy or had either a mild case of periodontitis (stage 1) or a low progression rate (grade A) as shown in Table 1, respectively. This can also be explained by the generally high prevalence of periodontal disease in this patient cohort [2,9]. Thus, the individuals in the current study suffered from their disease for several years (RA: 6.6 and AS: 11.0). During their years of rheumatic disease, all of them appear to have developed a periodontal disease, which is displayed in the existing periodontal disease burden in the cohort. It is controversially discussed whether this is simply an association or even a causal (bidirectional) relationship [3,4]. To further evaluate this potential relationship, this current study examined the PISA, which reflects the inflammatory periodontal burden of a patient by determining the sum of BOP-positive periodontal pockets [12,13]. This parameter is of clinical interest but might be affected by several factors during clinical recordings or by some conditions like smoking and systemic medications that may be able to modify the proportion of BOP-positive sites. The PISA has been reported to be related to periodontal disease severity but also to bacterial burden, e.g., *Porphyromonas gingivalis* [13]. While a previous clinical study failed to confirm associations between single clinical and/or microbiological parameters with disease-related factors in RA individuals [8], the PISA was expected to be more meaningful in this context. This is supported by Garner et al., who stated that PISA would be more promising than traditional periodontal measurements for detection of associations with rheumatic disease activity [18]. However, none of the rheumatic disease-related parameters was found to be associated with PISA in RA or AS patients, respectively. This appears somewhat surprising, especially because the disease activity of RA was found to be associated with the severity of periodontal disease [3,5]. Similarly, AS activity has been found to be associated with periodontal disease [9,10].

Until now, only one study has been available, which investigated PISA in RA individuals, showing associations with disease activity and swollen joints [14]. Thereby, this previous study showed associations with the clinical response to disease-modifying antirheumatic drugs, although only within a small group (n = 54) with a high diversity of participants [14]. This fact might lead to one potential explanation of the current study’s results: the heterogeneity of the study cohort, regarding disease activity and medication. This high heterogeneity within and between studies has already been highlighted in a recent systematic review and meta-analysis [4]. Regardless of this, however, the missing association between PISA and disease-related parameters in RA and AS in context of the recent literature underlines the complexity of the topic and the questionability of a bidirectional relationship of the diseases, as stated by Hussain et al. [3]. The role of disease-related medication especially appears to require further consideration in the future, as this might be a major influential factor on periodontitis severity [19,20]. 

In contrast to PISA, the number of remaining teeth showed several associations with disease activity, especially in the RA group in the current study. A higher prevalence of tooth loss in individuals suffering from RA has already been presumed in previous studies [8,21,22,23]. Furthermore, missing teeth were found to be a potential influential factor on proinflammatory cytokines in RA patients [24]. Additionally, a Japanese study reported tooth loss to be related to stronger RA therapies (medication), independent of the disease activity of RA [6]. The association of fewer remaining teeth with prednisolone therapy in the current study might support this. First, of course, tooth loss occurs because of advanced periodontal burden [25]. Therefore, the association between disease-related parameters and fewer remaining teeth could be an association with periodontal disease burden (especially long disease progress). However, tooth loss is a complex problem, potentially causing different effects. Nutrition is especially affected by the presence of remaining teeth, and tooth loss increases risk of malnutrition [26]. This might also increase RA disease activity, which is influenced by dietary factors as well [27]. Furthermore, tooth loss was found to affect muscle and handgrip strength in the general population [28], which also might indicate a complex interrelation between tooth loss and RA (and SA) activity. Furthermore, the occurrence of tooth loss as a potential consequence of periodontal bone loss might also affect the PISA results of the current study cohort. If patients lost many of their teeth, especially molars, their PISA might be lower, because there is simply less periodontal surface that can be inflamed. In the RA group, this effect can be observed (see Table 3). This could have affected the evaluation of potential associations between PISA and disease activity, as this is a contrary effect to tooth loss.

Altogether, the findings of the current study in the context of the available literature might suggest a more complex interrelationship between oral conditions and RA as well as SA and not, primarily, a bidirectional relationship on an etiopathogenetic basis. From the clinical perspective, the avoidance of tooth loss by including patients early in disease progress in a prevention-oriented dental care protocol might be recommendable.

Strengths and limitations: This is the first study which examined both patients with RA and AS regarding potential associations between PISA and remaining teeth with disease-related parameters. Skilled, experienced and calibrated dentists performed the clinical examinations under standardized conditions. Moreover, the sample size in the RA group was quite large, with 101 patients. However, no power calculation was performed, and therefore, the conclusions of the current study, especially on the AS group, need to be interpreted with caution. Similarly, the cut-off for the analysis of associations between PISA and remaining teeth with disease-related parameters was set by median, respectively. These values are not validated by the literature, making a comparison quite difficult. Thereby, it would have been possible to use the median for both cohorts (RA and AS) separately. However, for the current analysis, the median of the total cohort (n = 133) was chosen as the cut-off to increase the potential to draw meaningful conclusions. Nevertheless, this is a limitation of the current analysis and a kind of arbitrary value. For future studies, a cut-off value based on all available studies on PISA and/or tooth loss of patients with rheumatic disease might be determined and used for analysis. Furthermore, no information regarding the periodontal history of the patients, especially previous therapy, was available, which might have affected the PISA findings. Additionally, this might be of interest with regard to the reasons for tooth loss to ensure correct classification. Moreover, no radiographs were taken, and thus, the bone loss cannot be assessed. However, there was no justifying indication for X-ray diagnostics for the patients within the current study, so it was not possible to receive the respective radiographic information. The absence of a healthy control group can be seen as a limitation; however, the aim of this study was to examine potential associations between PISA and remaining teeth with disease-related factors, which would not be present in healthy individuals, respectively. Therefore, a healthy control group would not provide any benefit for answering the study question. Furthermore, the groups were quite heterogeneous regarding disease duration, disease activity and medication. This also limits the generalizability of the findings. The current study is limited by its cross-sectional design, whereby no causal conclusions can be made, and no long-term effects can be evaluated.

## 5. Conclusions

PISA is not associated with disease-related parameters in patients with RA and SA, while tooth loss is associated with disease activity, especially in RA individuals. The relation between oral conditions and RA and SA appears complex. Dental care to prevent tooth loss, ideally starting in the early disease progress, might be recommendable to positively influence oral health and disease activity in RA or SA patients.

## Figures and Tables

**Table 1:** Patient characteristics.

			RA [n = 101]	AS [n = 32]
Gender (male) [n (%)]			19 (18.8%)	18 (56.3%)
Age in years (MV ± SD) [median]			57.48 ± 10.03 (57)	48.37 ± 16.77 (46)
Smoking habits [n (%)]		smoker	20 (19.8%)	13 (40.6%)
		nonsmoker	81 (80.2%)	19 (59.4%)
Disease duration in years (MV ± SD)			6.60 ± 8.49	11.00 ± 12.18
Number of swollen joints (MV ± SD)			2.77 ± 3.57	0.34 ± 1.41
Number of painful joints (MV ± SD)			6.01 ± 6.79	1.97 ± 3.76
Morning stiffness in min (MV ± SD)			31.53 ± 36.70	27.25 ± 29.51
CRP (mg/L) (MV ± SD)			5.24 ± 8.47	6.29 ± 11.59
Disease activity (DAS28-ESR) (MV ± SD)			3.44 ± 1.42	-
aCCP (U/mL)			84.69 ± 132.44	-
HLA-B27 positive			-	23 (72%)
BASDAI (MV ± SD)			-	3.40 ± 2.41
BASMI (MV ± SD)			-	2.59 ± 1.74
BASFI (MV ± SD)			-	2.53 ± 2.01
BASG (MV ± SD)			-	4.06 ± 2.93
Rheumatism medication	Prednisolone		83 (82.2%)	6 (18.7%)
	Methotrexate		55 (54.5%)	2 (6.3%)
	Biologicals		33 (32.7%)	20 (62.5%)

(RA: rheumatoid arthritis group, AS: ankylosing spondylitis group, n: number of patients, MV: mean value, SD: standard deviation, CCP: cyclic citrullinated peptide, CRP: c-reactive protein, HLA: human leukocyte antigen, BASDAI: Bath Ankylosing Spondylitis Disease Activity Index, BASMI: Bath AS Metrology Index, BASFI: Bath Ankylosing Spondylitis Functional Index, BASG: Bath Ankylosing Spondylitis Patient Global Score).

**Table 2:** Dental and periodontal findings of the investigated patients.

		RA Group (n = 101)	AS Group (n = 32)	*p*-Value
Number of remaining teeth (MV ± SD)		22.85 ± 4.26	24.34 ± 5.47	**<0.01**
Number of remaining molars/premolars (MV ± SD)		11.43 ± 3.64	12.91 ± 3.86	**0.01**
Number of remaining front teeth (MV ± SD)		11.43 ± 1.24	11.44 ± 1.95	0.24
Number of teeth CAL ≥ 5 mm (MV ± SD)		8.65 ± 6.17	4.31 ± 4.86	**<0.01**
Number of teeth PPD ≥ 5 mm (MV ± SD)		6.92 ± 5.86	5.38 ± 5.71	**0.02**
BOP (MV ± SD)		33.55 ± 17.58	45.09 ± 21.27	**<0.01**
Periodontitis stage [n (%)]	I	0	0	**0.04**
	II	9 (9%)	6 (18.8%)	
	III	46 (45.5%)	19 (59.4%)	
	IV	46 (45.5%)	7 (21.8%)	
Periodontitis grade [n (%)]	B	81 (80.2%)	19 (59.4%)	**0.03**
	C	20 (19.8%)	14 (40.6%)	
PISA in mm^2^ (MV ± SD)		564.69 ± 397.20	614.43 ± 357.12	0.35

(PPD: periodontal probing depths, CAL: clinical attachment loss, BOP: bleeding on probing, n: number of patients, MV: mean value, SD: standard deviation, PISA: periodontal inflamed surface area, significant findings (*p* < 0.05) are highlighted in bold).

**Table 3:** Association between PISA results and disease-related parameters in RA individuals.

			PISA		*p*-Value
			≤504.89 (n = 54)	>504.89 (n = 47)	
Gender (male) [n (%)]			13 (24.1%)	6 (12.8%)	0.20
Age in years (MV ± SD)			57.89 ± 10.03	57.00 ± 10.12	0.55
Smoking habits [n (%)]		smoker	6 (11.1%)	33 (70.2%)	**0.03**
		nonsmoker	48 (88.9%)	14 (29.8%)	
Number of remaining teeth (MV ± SD)			22.04 ± 4.59	23.79 ± 3.66	**0.05**
Disease duration in years (MV ± SD)			6.20 ± 8.42	7.06 ± 8.63	0.57
Number of swollen joints (MV ± SD)			3.21 ± 4.17	2.28 ± 2.71	0.47
Number of painful joints (MV ± SD)			6.04 ± 7.13	5.98 ± 6.47	0.64
Morning stiffness in min (MV ± SD)			33.19 ± 33.82	29.64 ± 40.04	0.12
CRP (mg/L) (MV ± SD)			6.71 ± 11.11	3.54 ± 2.86	0.98
Disease activity (DAS28-ESR) (MV ± SD)			3.60 ± 1.53	3.26 ± 1.27	0.29
aCCP (U/mL)			74.51 ± 119.10	96.39 ± 146.71	0.81
Rheumatism medication	Prednisolone		47 (87%)	36 (76.6%)	0.29
	Methotrexate		32 (59.3%)	23 (48.9%)	0.32
	Biologicals		17 (31.5%)	16 (34%)	0.83

(n: number of patients, MV: mean value, SD: standard deviation, CCP: cyclic citrullinated peptide, CRP: c-reactive protein, DAS: disease activity score, significant findings (*p* < 0.05) are highlighted in bold).

**Table 4:** Association between PISA results and disease-related parameters in AS individuals.

			PISA		*p*-Value
			≤504.89 (n = 13)	>504.89 (n = 19)	
Gender (male) [n (%)]			7 (53.8%)	11 (57.9%)	0.99
Age in years (MV ± SD)			43.54 ± 16.54	51.68 ± 16.55	0.08
Smoking habits [n (%)]		smoker	8 (61.5%)	11 (57.9%)	0.99
		nonsmoker	5 (38.5%)	8 (42.1%)	
Number of remaining teeth (MV ± SD)			23.15 ± 7.78	25.16 ± 3.08	0.97
Disease duration in years (MV ± SD)			6.15 ± 5.43	14.32 ± 14.40	0.13
Number of swollen joints (MV ± SD)			0.31 ± 1.11	0.37 ± 1.61	0.82
Number of painful joints (MV ± SD)			1.69 ± 4.61	2.16 ± 3.18	0.18
Morning stiffness in min (MV ± SD)			25.54 ± 39.77	28.42 ± 21.02	0.18
CRP (mg/L) (MV ± SD)			9.66 ± 16.97	3.98 ± 5.05	0.39
HLA-B27 positive			9 (69.2%)	14 (73.7%)	0.99
BASDAI (MV ± SD)			2.81 ± 2.47	3.80 ± 2.35	0.16
BASMI (MV ± SD)			2.35 ± 1.97	2.76 ± 1.59	0.34
BASFI (MV ± SD)			2.19 ± 2.47	2.75 ± 1.82	0.18
BASG (MV ± SD)			3.42 ± 2.85	4.50 ± 2.99	0.26
Rheumatism medication	Prednisolone		3 (23.1%)	2 (10.5%)	0.47
	Methotrexate		0	2 (10.5%)	0.50
	Biologicals		9 (69.2%)	11 (57.9%)	0.71

(n: number of patients, MV: mean value, SD: standard deviation, CRP: c-reactive protein, HLA: human leukocyte antigen, BASDAI: Bath Ankylosing Spondylitis Disease Activity Index, BASMI: Bath AS Metrology Index, BASFI: Bath Ankylosing Spondylitis Functional Index, BASG: Bath Ankylosing Spondylitis Patient Global Score).

**Table 5:** Association between number of remaining teeth and disease-related parameters in RA individuals.

			Number of Remaining Teeth		*p*-Value
			≤24 (n = 61)	>24 (n = 40)	
Gender (male) [n (%)]			15 (24.6%)	4 (10%)	0.08
Age in years (MV ± SD)			60.23 ± 9.02	53.28 ± 10.14	**<0.01**
Smoking habits [n (%)]		smoker	14 (23%)	6 (15%)	0.45
		nonsmoker	47 (77%)	34 (85%)	
Disease duration in years (MV ± SD)			7.16 ± 9.63	5.75 ± 6.38	0.81
Number of swollen joints (MV ± SD)			2.92 ± 3.84	2.55 ± 3.15	0.69
Number of painful joints (MV ± SD)			6.95 ± 7.63	4.60 ± 5.07	0.12
Morning stiffness in min (MV ± SD)			30.97 ± 30.71	32.40 ± 44.74	0.43
CRP (mg/L) (MV ± SD)			6.52 ± 10.16	3.27 ± 4.32	**0.02**
Disease activity (DAS28-ESR) (MV ± SD)			3.79 ± 1.39	2.91 ± 1.31	**<0.01**
aCCP (U/mL)			76.29 ± 128.35	97.51 ± 139.10	0.92
Rheumatism medication	Prednisolone		56 (91.8%)	27 (67.5%)	**<0.01**
	Methotrexate		32 (52.5%)	23 (57.5%)	0.69
	Biologicals		20 (32.8%)	13 (32.5%)	0.99

(n: number of patients, MV: mean value, SD: standard deviation, CCP: cyclic citrullinated peptide, CRP: c-reactive protein, significant findings (*p* < 0.05) are highlighted in bold).

**Table 6:** Association between remaining teeth and disease-related parameters in AS individuals.

			Number of Remaining Teeth		*p*-Value
			≤24 (n = 12)	>24 (n = 20)	
Gender (male) [n (%)]			7 (58.3%)	11 (55%)	0.99
Age in years (MV ± SD)			57.67 ± 16.02	42.80 ± 14.94	0.02
Smoking habits [n (%)]		smoker	7 (58.3%)	12 (60%)	0.99
		nonsmoker	5 (41.7%)	8 (40%)	
Disease duration in years (MV ± SD)			12.83 ± 13.04	9.90 ± 11.84	0.57
Number of swollen joints (MV ± SD)			0.58 ± 2.02	0.20 ± 0.89	0.68
Number of painful joints (MV ± SD)			1.67 ± 3.03	2.15 ± 4.21	0.71
Morning stiffness in min (MV ± SD)			27.08 ± 20.39	27.35 ± 34.35	0.54
CRP (mg/L) (MV ± SD)			2.84 ± 3.41	8.35 ± 14.16	0.12
HLA-B27 positive			7 (58.3%)	16 (80%)	0.24
BASDAI (MV ± SD)			3.86 ± 2.43	3.12 ± 2.42	0.41
BASMI (MV ± SD)			3.52 ± 1.75	2.04 ± 1.52	**0.02**
BASFI (MV ± SD)			2.86 ± 2.23	2.33 ± 2.03	0.38
BASG (MV ± SD)			4.25 ± 3.00	3.95 ± 2.97	0.80
Rheumatism medication	Prednisolone		4 (33.3%)	1 (5%)	0.08
	Methotrexate		1 (8.3%)	1 (5%)	0.99
	Biologicals		8 (66.7%)	12 (60%)	0.99

(n: number of patients, MV: mean value, SD: standard deviation, CRP: c-reactive protein, HLA: human leukocyte antigen, BASDAI: Bath Ankylosing Spondylitis Disease Activity Index, BASMI: Bath AS Metrology Index, BASFI: Bath Ankylosing Spondylitis Functional Index, BASG: Bath Ankylosing Spondylitis Patient Global Score, significant findings (*p* < 0.05) are highlighted in bold).

## Data Availability

Data are available from the corresponding author upon reasonable request.
